# *In silico* Docking Studies of Fingolimod and S1P_1_ Agonists

**DOI:** 10.3389/fphar.2020.00247

**Published:** 2020-03-10

**Authors:** Alexander Marciniak, Sara M. Camp, Joe G. N. Garcia, Robin Polt

**Affiliations:** ^1^Department of Chemistry and Biochemistry, The University of Arizona, Tucson, AZ, United States; ^2^Department of Medicine, The University of Arizona, Tucson, AZ, United States

**Keywords:** S1P_1_, GPCR, FTY720, Molecular Operating Environment, *in silico* modeling, sphingosine phosphate

## Abstract

The sphingosine-1-phosphate receptor 1 (S1P_1_), originally the endothelial differentiation gene 1 receptor (EDG-1), is one of five G protein–coupled receptors (GPCRs) S1P_1__–__5_ that bind to and are activated by sphingosine-1-phosphate (S1P). The lipid S1P is an intermediate in sphingolipid homeostasis, and S1P_1_ is a major medical target for immune system modulation; agonism of the receptor produces a myriad of biological responses, including endothelial cell barrier integrity, chemotaxis, lymphocyte trafficking/targeting, angiogenesis, as well as regulation of the cardiovascular system. Use of *in silico* docking simulations on the crystal structure of S1P_1_ allows for pinpointing the residues within the receptor’s active site that actively contribute to the binding of S1P, and point to how these specific interactions can be exploited to design more effective synthetic analogs to specifically target S1P_1_ in the presence of the closely related receptors S1P_2_, S1P_3_, S1P_4_, and S1P_5_. We examined the binding properties of the endogenous substrate as well as a selection of synthetic sphingosine-derived S1P_1_ modulators of S1P_1_ with *in silico* docking simulations using the software package Molecular Operating Environment^®^ (MOE^®^). The modeling studies reveal the relevance of phosphorylation, i.e., the presence of a phosphate or phosphonate moiety within the substrate for successful binding to occur, and indicate which residues are responsible for S1P_1_ binding of the most prominent sphingosine-1-phosphate receptor (S1PR) modulators, including fingolimod and its structural relatives. Furthermore, trends in steric preferences as for the binding of enantiomers to S1P_1_ could be observed, facilitating future design of receptor-specific substrates to precisely target the active site of S1P_1_.

## Introduction

With over 800 types encoded alone in the human genome, G protein–coupled receptors (GPCRs) constitute the largest family of 7-transmembrane (7TM) domain receptors ubiquitously expressed in all eukaryotic organisms and are responsible for numerous biological processes as intercellular signaling gateways (Pierce et al., 2002; Fredriksson et al., 2003). Originally termed the endothelial differentiation gene (EDG), sphingosine-1-phosphate receptor (S1PR) class GPCRs rely on the phosphorylated sphingoid base sphingosine-1-phosphate (S1P) for agonism (activation), and are involved in a multitude of pathophysiological processes as they regulate cellular barrier integrity, differentiation and proliferation, cell migration, angiogenesis, as well as immunity (Garcia et al., 2001; Matloubian et al., 2004; Spiegel and Weinstein, 2004; Heng et al., 2013; Bigaud et al., 2014; Camp et al., 2020). This involvement with diverse diseases and syndromes makes GPCRs a major medicinal drug target with approximately 40% of all therapeutic agents being developed to target this class of receptors (Hauser et al., 2017; Santos et al., 2017). Of the five known types of S1PRs (S1P_1__–__5_), S1P_1_ (EDG-1) is of particular interest since it has been shown to be the primary vascular barrier-regulatory receptor (Garcia et al., 2001; Dudek et al., 2004; McVerry et al., 2004; Peng et al., 2004; Sammani et al., 2010). Importantly, autoimmune diseases utilize S1P–induced agonism of S1P_1_ for expression and progression of symptoms (Lee et al., 1998; Toman and Spiegel, 2002; Brinkmann, 2007). As binding efficiency and receptor specificity profoundly dictate the efficacy of protein–targeting drugs, synthetic small molecule agents developed to target S1P_1_ require distinct structural features to successfully mimic endogenous S1P and competitively bind to the receptor’s active site. Developed in 1992 and approved by the FDA in 2010, FTY720 (Fingolimod, Gilenya^®^) has been shown to be vascular protective in the acute clinical setting (Peng et al., 2004; Camp et al., 2009; Wang et al., 2011), and has been adopted for oral administration to treat relapsing-remitting multiple sclerosis (RRMS; Chiba, 2005; Adachi and Chiba, 2007; Brinkmann et al., 2010; Baer et al., 2018). However, due to adverse effects arising from S1PR promiscuity and because of the prodrug character of fingolimod, the search for better immunosuppressive agents with enhanced cellular stability and greater receptor specificity is still a current research topic (Thomson, 2006; Cohen et al., 2010). The successful co-crystallization of S1P_1_ bound to the synthetic antagonist ML056 (W146) by Hanson et al. (2012) has allowed access to the crystal structure of S1P_1_ for *in silico* analyses of the binding behavior of known, as well as novel substrates within the active site of the receptor (Hanson et al., 2012).

## Materials and Methods

### Software and Crystal Structure File

All *in silico* molecular mechanics docking simulations were conducted using the software package Molecular Operating Environment^®^ (MOE^®^) version 2018.01 from Chemical Computing Group (CCG)^1^. The crystal structure of S1P_1_ was obtained from the Protein Data Bank (PDB) file 3V2Y, giving the structure of S1P_1_ bound to the antagonist ML056 (W146, Table 1) at 2.8 Å resolution, as reported by Hanson et al. (2012). The crystal structure of S1P_1_ was validated using ERRAT, with the optimal obtained quality factor exceeding 95% (see Supplementary Material)^2^. The data used did not contain external solvent outside of S1P_1_. However, co-crystallized internal water molecules were present within the receptor depicted and were taken into account in all calculations performed.

**TABLE 1:** Substrates in ionization states as shown docked to S1P_1_ (Camp et al., 2009; Lu et al., 2009).

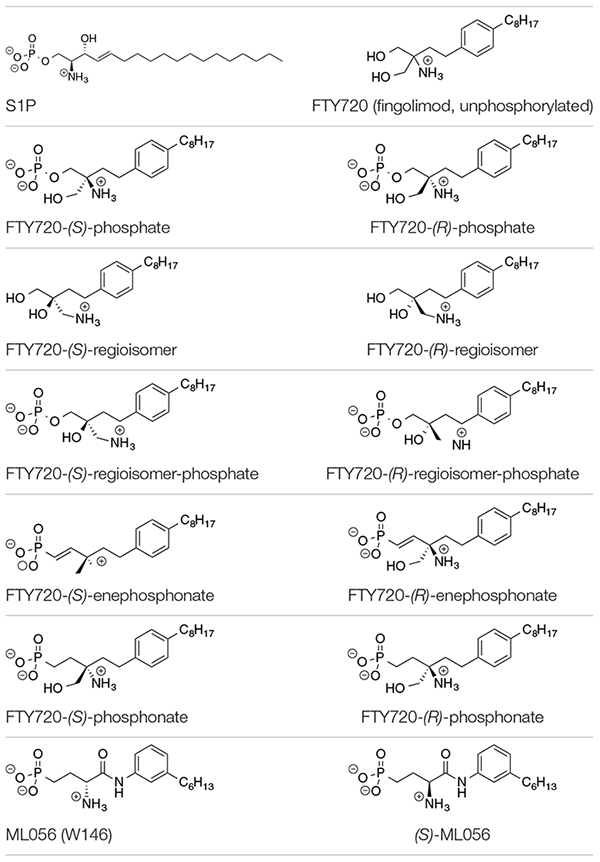

### Ligands and Ionization States

The compounds for the docking simulations were crafted via MOE Builder^®^ and manually inserted into the active site of S1P_1_ extracted from PDB file 3V2Y. Table 1 depicts the ligands docked *in silico* to S1P_1_. The selection of ligands chosen to be subjected to the docking experiments comprises of the most prominent immunosuppressant fingolimod, its regioisomers, as well as structural phosphonate derivatives. In order to examine the impact of phosphorylation on the binding behavior to S1P_1_, both phosphorylated and unphosphorylated versions of agents known to exhibit bioactivity only upon *in vitro* and/or *in vivo* phosphorylation were included (Camp et al., 2009; Lu et al., 2009; Mathew et al., 2011; Wang et al., 2014, 2015).

All substrates containing primary amines and phosphoester or phosphonate moieties were set with ionization states expected for S1P at physiological pH. Computational p*K*_*a*_ results indicate S1P binds to S1P_1_ with an overall charge of −1; the protonated amino group carries a +1 charge while the phosphate exists in fully deprotonated state with a charge of −2, stabilized by physiological pH, intramolecular hydrogen bonding of the phosphate with the protonated hydroxyl group of S1P, as well as by the microenvironment created by the residues of the active site within S1P_1_ (Naor et al., 2007). This ionization state was applied to all substrates in equivalent manner, with primary amino groups being protonated, obtaining a charge of +1, and phosphates and phosphonates being fully deprotonated, carrying a −2 charge.

### Simulation Setup and Docking Parameters

Manual substitution of the antagonist ML056 (W146) in the active site of S1P_1_ with the compounds to be docked to the receptor was the starting point of the simulations. Once positioned in the receptor’s active site, energy minimization of the substrate’s structure was performed to fully embed the freshly placed ligand within S1P_1_ and to ensure no overlaps of ligand atoms with receptor residues would occur. The ligand was selected and the docking experiments were then executed with parameters set as summarized in Table 2. Most settings were kept in MOE^®^ standard; the triangle matcher method was used as the placement phase with London dG scoring. In order to obtain realistic substrate–receptor interactions with resulting conformations, the induced fit model was set for the refinement method with GBVI/WSA dG scoring. No distinct pharmacophores were defined in MOE Pharmacophore Editor^®^ as the purpose of the experiments was to observe which moieties, their constellation, and their presence or absence would trigger or inhibit effective binding to the active site of S1P_1_ with minimal restrictions during *in silico* docking. The docking experiment of each substrate was performed 100 times and the 25 lowest energy conformations (poses) were recorded in order of increasing potential energy of the conformer in kcal/mol. For specific binding analysis, the structure of the energetically lowest S1P_1_–substrate conformation was visualized and evaluated for polar, hydrophobic, and steric interactions between the ligand atoms or moieties and specific residues of S1P_1_.

**TABLE 2:** MOE^®^ docking simulation parameters.

**MOE^®^ docking parameter**	**Setting**
Receptor	“MOE (receptor+solvent)”
Site	“Ligand atoms”
Pharmacophore	“None”
Ligand	“MOE (ligand atoms)”
Placement	“Triangle matcher”
Placement score	“London dG”
Refinement	“Induced fit”
Refinement score	“GBVI/WSA dG”
Poses	“25/100”

## Results

### Binding of S1P, ML056 (W146), and *(S)* Enantiomer of ML056 to S1P_1_

The lowest value for the potential energy of a docked conformer of S1P to the active site of S1P_1_ was −351.7 kcal/mol (Table 3). This value then served as the qualitative standard for all further docking energies obtained from synthetic substrates in order to determine satisfactory or poor binding quality. Almost all of the recorded 25 minimal energy values showed just small increases in energy, with the 24th energy value still being at −331.9 kcal/mol (see Supplementary Material), indicating continuously stable binding of the endogenous ligand to its receptor.

**TABLE 3:** Energy of conformer values of docked substrates to S1P_1_.

	**Lowest calculated energy**
**Compound**	**of conformer (kcal/mol)**
FTY720-*(R)*-phosphate	–384.8
FTY720-*(S)*-phosphate	–383.3
FTY720-*(R)*-regioisomer-phosphate	–365.7
FTY720-*(S)*-regioisomer-phosphate	–363.1
S1P	–351.7
FTY720-*(S)*-phosphonate	–339.9
FTY720-*(R)*-phosphonate	–338.6
ML056 (W146)	–331
FTY720-*(R)*-enephosphonate	–317.1
FTY720-*(S)*-enephosphonate	–314.3
*(S)*-ML056	–266.2
FTY720 (unphosphorylated)	–13.5
FTY720-*(R)*-regioisomer (unphosphorylated)	–1.9
FTY720-*(S)*-regioisomer (unphosphorylated)	–0.8

**FIGURE 1:**
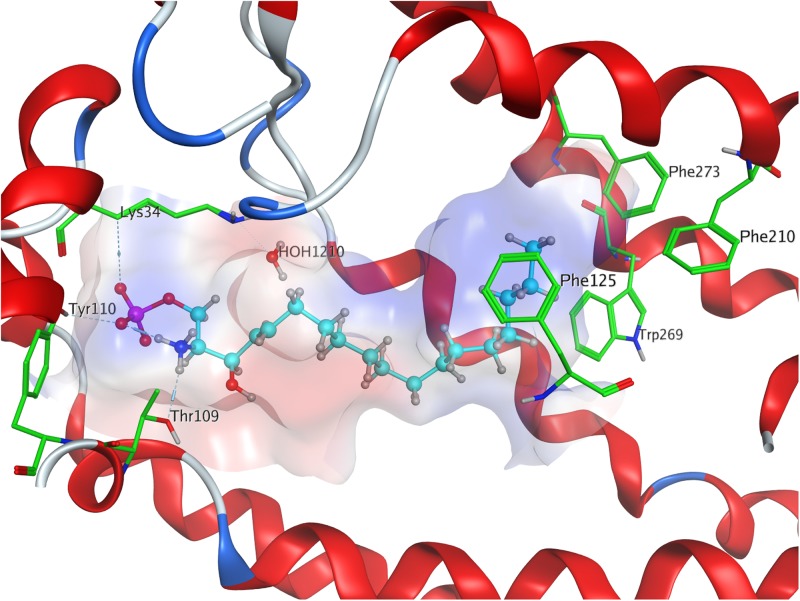
Energetically lowest calculated conformation of S1P in S1P_1_.

Both intra- and intermolecular interactions contribute to the low value recorded; one of the deprotonated oxygen atoms of the phosphate moiety forms a hydrogen bond with the hydroxyl proton of Y110^2.63^, while the other deprotonated oxygen of the phosphate hydrogen-bonds intramolecularly with the protonated amine of S1P. The latter simultaneously forms another hydrogen bond with residue T109. Additionally, the oxygen of the phosphoryl bond interacts with the alkyl chain of K34, further fixing the ligand’s head group in position of the active site. The hydrophobic alkyl chain of S1P inserts into an aromatic binding pocket formed by residues F125^3.33^, F210^5.47^, and F273^6.52^ between the transmembrane helices of S1P_1_, also visibly affecting the conformation of W269^6.48^ (Figure 1).

For reference purposes, the co-crystallized S1P_1_ antagonist ML056 (W146) from the original PDB file 3V2Y was also subjected to the same docking all other substrates underwent. ML056 (W146) scored a minimal energy of −331 kcal/mol (Table 3), with only a slight increase in conformational energy for the 25th recorded conformer, −310.9 kcal/mol (see Supplementary Material).

**FIGURE 2:**
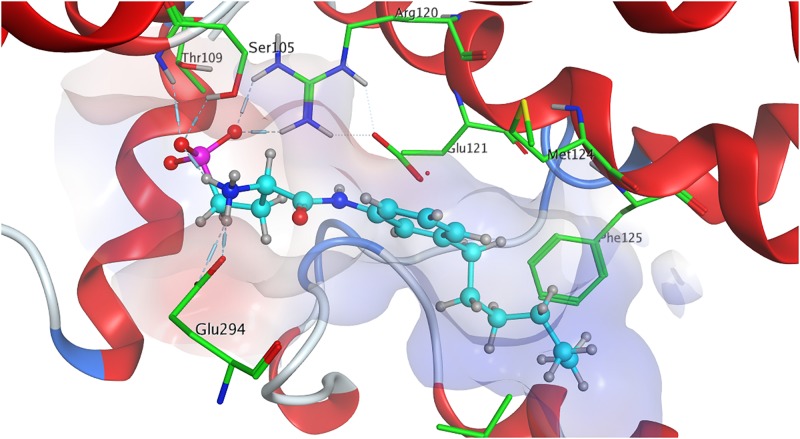
Energetically lowest calculated conformation of ML056 (W146) in S1P_1_.

The strong binding of ML056 (W146) to S1P_1_ results from multiple hydrogen bonds fixing the phosphonate of the substrate’s head group to polar residues of the active site of S1P_1_; T109 donates to both deprotonated oxygen atoms of the phosphonate, while S105^2.64^ and G106 form further hydrogen bonds with the phosphoryl oxygen of the phosphonate. Residues R120^3.28^ and E121^3.29^ form multiple hydrogen bonds, both donating to one of the deprotonated phosphonate oxygen of ML056 (W146) and interacting with each other, locking the residues’ constellation rigidly into place. Residue E121^3.29^ also acts as an acceptor for the substrate’s amid hydrogen. The protonated amine of ML056 (W146) also interacts with E294^7.36^, donating one of its hydrogen atoms to the deprotonated residue. The hydrophobic C6 chain of the S1P_1_ antagonist is in vicinity of F125^3.33^, M124^3.32^, and L272^6.51^ for non-polar interaction (Figure 2).

For comparison, the *in silico* generated *(S)* enantiomer of ML056 (in the following referred to as *(S)*-ML056, Table 1) was also docked to S1P_1_ with identical prerequisite and simulation settings. The minimal energy of conformer obtained was −266.2 kcal/mol, being significantly higher than the co-crystallized *(R)* enantiomer (Table 3). The polar headgroup of the antagonist’s *(S)* enantiomer formed multiple hydrogen bonds with residues Y29, K34, and R120^3.28^; all three residues acting as hydrogen bond donors to the deprotonated oxygen atoms of the phosphonate. Lysine residue K34 also formed a triangle bridge with a water molecule trapped within the active site’s cavity to further bind to the phosphoryl oxygen atom of the substrate’s headgroup. Residues N101^2.60^ and E121^3.29^ also acted as hydrogen bond acceptors of the protonated amino group of *(S)*-ML056. However, the non-polar residue F125^3.33^ sterically interfered with the direct interaction of these polar residues, showing the stereochemical relevance for proper substrate–receptor fitting (see Supplementary Material).

### Binding of FTY720 and Its *(S)*- and *(R)*-Phosphates to S1P_1_

FTY720 (fingolimod) was prepared in trifold version for the docking experiment to S1P_1_; the unphosphorylated drug was docked into the active site of S1P_1_, followed by the docking of its respective *(S)*- and *(R)*-phosphates for direct comparison.

**FIGURE 3:**
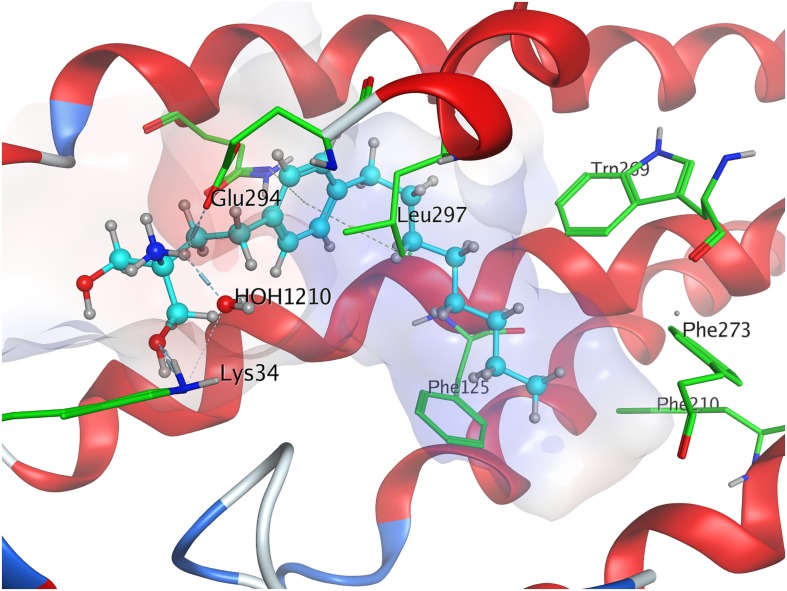
Energetically lowest calculated conformation of unphosphorylated FTY720 in S1P_1_.

When docked to S1P_1_, unphosphorylated fingolimod was found to achieve a comparably high minimal energy of conformer value of −13.5 kcal/mol, with slight but steady energy increase of the subsequent 24 most stable conformers calculated (Table 3; see Supplementary Material). The pro-*(R)* hydroxyl group of FTY720 forms a hydrogen bond with the amine of K34, which, in turn, coordinates to a water molecule trapped within the active site of S1P_1_. This water molecule also hydrogen-binds to the protonated amine of fingolimod, forming an intermolecular bridge between its hydroxyl group and its amine moiety. The protonated amine of FTY720 furthermore hydrogen-binds to the deprotonated carboxyl oxygen of E294^7.36^. The aromatic core of fingolimod was found to be trapped between L297^7.39^ and N101^2.60^, while the drug’s alkyl tail is embedded between the transmembrane helices’ residues F125^3.33^, F210^5.47^, F273^6.52^, and W269^6.48^ (Figure 3).

**FIGURE 4:**
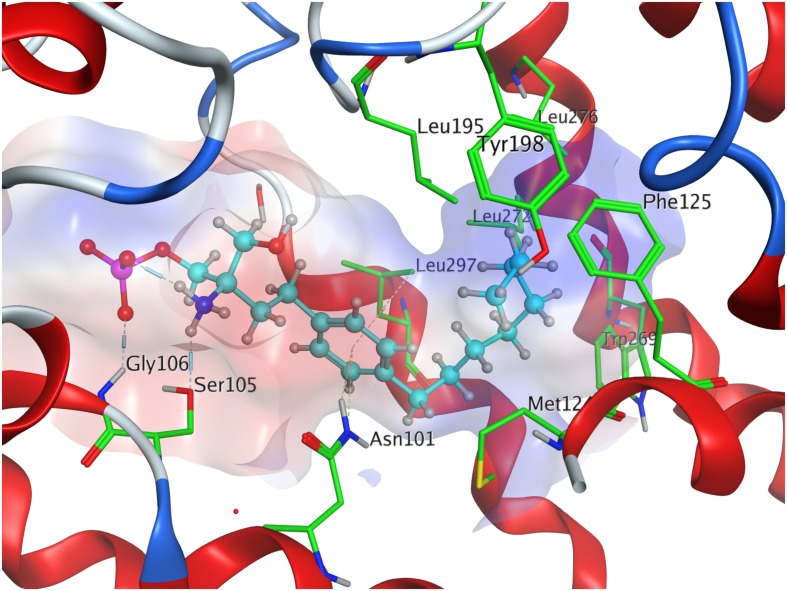
Energetically lowest calculated conformation of FTY720-*(S)*-phosphate in S1P_1_.

Phosphorylation of the substrate leads to substantial gain in energy upon interaction with the active site of S1P_1_; the *(S)*-phosphate of FTY720 was calculated to obtain a minimal energy of conformer value of −383.3 kcal/mol, significantly exceeding the binding affinity registered at S1P docking (Table 3). The rise in energetical values observed in the subsequent 24 optimized poses of fingolimod-*(S)*-phosphate in S1P_1_ was relatively small, with the 25th value still being maintained at −360.2 kcal/mol (see Supplementary Material). The phosphoryl of the ligand interacts with the alkyl chain of K34 while one of the phosphate’s deprotonated oxygen atoms forms a hydrogen bond with the acidic proton of Y110^2.63^. The other deprotonated oxygen of the phosphate engages in an intramolecular hydrogen bond with the substrate’s protonated amino group. Fingolimod’s C8 chain is embedded between helical residues Y98^2.57^, M124^3.32^, F125^3.33^, and W269^6.48^ (Figure 4).

**FIGURE 5:**
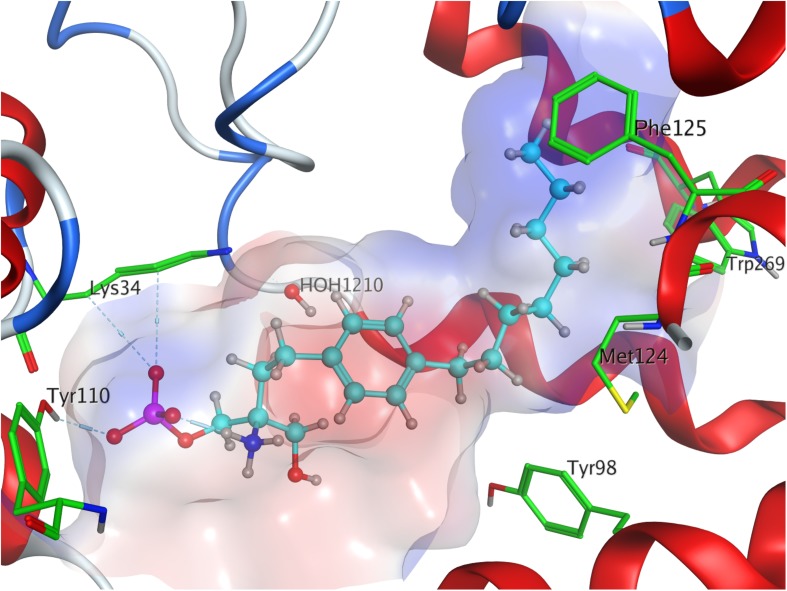
Energetically lowest calculated conformation of FTY720-*(R)*-phosphate in S1P_1_.

The docking simulation of the *(R)* enantiomer to the active site of S1P_1_ gave the lowest recorded minimal energy of conformer value of −384.8 kcal/mol with a minor energy increase of the following 24 subsequent conformers, ranging up to −362.1 kcal/mol for the 25th conformer (Table 3; see Supplementary Material). When docked into S1P_1_, the *(R)*-phosphate’s phosphoryl oxygen develops a hydrogen bond with the amide proton of G106 while the deprotonated oxygen of the phosphate intramolecularly hydrogen-bind to the protonated amino group of fingolimod. This amino group also interacts with S105^2.64^, while the aromatic core of the ligand is held in place by residues N101^2.60^ and L297^7.39^. The alkyl tail of fingolimod-*(R)*-phosphate was found to insert in a densely aligned pocket formed by transmembrane residues M124^3.32^, F125^3.33^, L195, Y198, W269^6.48^, L272^6.51^, and L276 (Figure 5).

### Binding of FTY720-*(S)*- and *(R)*-Regioisomers and Their Phosphates to S1P_1_

The docking experiments of the *(S)* and *(R)* regioisomers of fingolimod were also conducted with the compounds being in their unphosphorylated as well as phosphorylated states. As before, the lack of phosphate renders the ligands in stressed conformations within the active site of S1P_1_, yielding relatively high energy of conformer values in the docking simulations; the minimal calculated values for both unphosphorylated enantiomers in the receptor are close to 0 kcal/mol, indicating a strong drive toward active dissociation of the substrate–enzyme complex rather than binding. The energetic results of subsequent optimized conformers quickly exceed the threshold of 0 kcal/mol, reaching positive energy values (see Supplementary Material).

With a minimal conformational energy value of −1.918 kcal/mol (Table 3), the unphosphorylated *(R)*-regioisomer’s tertiary hydroxyl group’s hydrogen donates to a hydrogen bond with the oxygen of S105^2.64^, while the protonated secondary amine of the residue of R120^3.28^ forms another hydrogen bond with respective hydroxyl’s oxygen. The aromatic core of the ligand interacts with L297^7.39^ and N101^2.60^ and its alkyl chain strongly bent between hydrophobic transmembrane residues M124^3.32^, F125^3.33^, F210^5.47^, and in some more distance F273^6.52^.

The unphosphorylated *(S)* regioisomer of FTY720 displays the highest minimized energy (worst binding) of −0.8 kcal/mol (Table 3). Its protonated amine forms an intramolecular hydrogen bond with the oxygen of the regioisomer’s primary hydroxyl group and donates to another hydrogen bond with the oxygen of T109. The tertiary hydroxyl group of the ligand coordinates a water molecule in the active site of S1P1, which is further hydrogen-bound to the primary amine of K34. The alkyl tail of the ligand resides between F125^3.33^, F210^5.47^, F273^6.52^, and L272^6.51^ and L276.

The presence of a phosphate results in a significant drop in the energetics (better binding) of the formed fingolimod regioisomer conformers within the active site of S1P_1_; the docking simulation of the *(R)* regioisomer phosphate of FTY720 yielded a minimal value of −365.7 kcal/mol (Table 3) with solely a slight increase in potential energy up to −346.3 kcal/mol with the 24th substrate–receptor conformer simulated (see Supplementary Material). The phosphate actively participates in the fixation of the polar head group of the ligand to the hydrophilic cavity of the active site of S1P_1_; the phosphoryl oxygen of the ligand’s phosphate acts as the acceptor of a hydrogen bond donated by the hydroxyl group of Y110^2.63^ while the deprotonated oxygen atoms of the phosphate coordinate intramolecularly with both the protonated amine of the substrate as well as its hydroxyl group. The latter also exhibits another hydrogen bond to the amino group of K34. The C8 chain of the substrate aligns with the hydrophobic residues Y98^2.57^, M124^3.32^, W269^6.48^, and V301^7.42^.

FTY720-*(S)*-regioisomer-phosphate was docked to S1P_1_ yielding an optimized energy of conformer value of −363.1 kcal/mol (Table 3). The next 23 conformers recorded only reach a marginal energetic increase up to −343.9 kcal/mol (see Supplementary Material), supporting the stability of this ligand’s binding. In the active site of S1P_1_, the amide hydrogen of G106 donates to a hydrogen bond with the phosphoryl oxygen of the phosphate, which also forms an intramolecular hydrogen bond with the substrate’s protonated amino group. The latter furthermore locks onto S105^2.64^. The regioisomer’s hydroxyl group participates in further hydrogen bonding, as it acts as an acceptor of the protonated amine of K34. The aromatic center of the substrate is again locked in position by residues L297^7.39^ and N101^2.60^, so the hydrophobic chain can insert in the helical pocket formed by transmembrane residues F125^3.33^, F210^5.47^, W269^6.48^, and L272^6.51^.

### Binding of FTY720-*(S)*- and *(R)*-Phosphonates to S1P_1_

With the installation of a phosphonate moiety instead of the phosphoester generated *in vivo* in the case of fingolimod, Lu et al. (2009) created an analog of the FDA–approved immunosuppressant that withstands phosphatase cleavage, resulting in greater cellular stability of the agent. In our *in silico* docking analyses, the phosphonates proved to act very similarly to the conventional fingolimod phosphates, resulting in comparable energetic values for their optimized conformers within the active site of S1P_1_.

**FIGURE 6:**
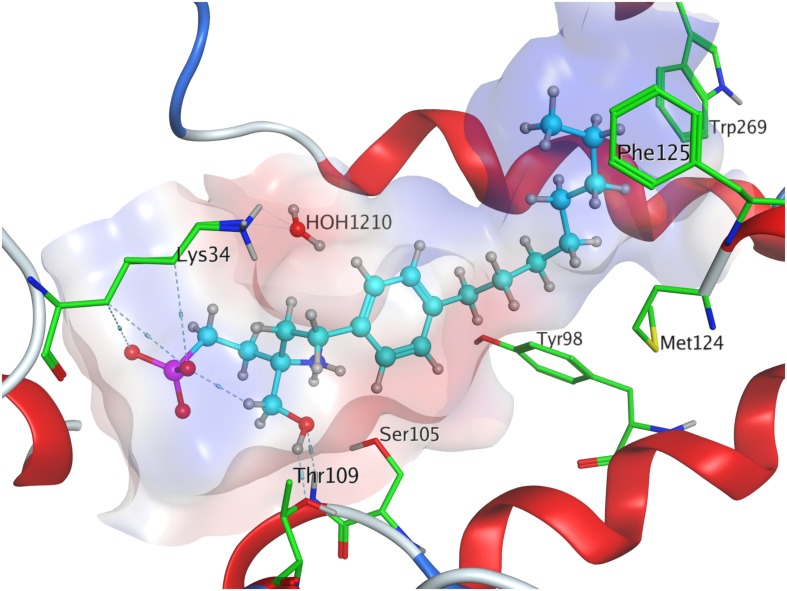
Energetically lowest calculated conformation of FTY720-*(S)*-phosphonate in S1P_1_.

Fingolimod’s *(S)*-phosphonate analog reaches after docking optimization a conformational energetic minimum of −339.9 kcal/mol (Table 3) with only a minor increase in potential energies of the remaining recorded conformers with values up to −319.2 kcal/mol (see Supplementary Material). The phosphoryl oxygen and one deprotonated oxygen of the phosphonate interact with the alkyl chain of K34. The hydroxyl group of the ligand forms a hydrogen bond with the amide proton of S105^2.64^ while the proton of same hydroxyl group of the substrate donates to another hydrogen bond with the oxygen of T109. The alkyl chain of FTY720-*(S)*-phosphonate firmly inserts between M124^3.32^, F125^3.33^, W269^6.48^, and Y98^2.57^ with slightly greater distance (Figure 6).

Docking of the *(R)*-phosphonate to the active cavity of S1P_1_ gave a minimal conformational energy value of −338.6 kcal/mol (Table 3). The increase in all subsequent recorded conformers is almost neglectable, reaching a maximum energy of conformer value of −318.3 kcal/mol with the 25th recorded substrate–enzyme conformer (see Supplementary Material). In the *(R)*-phosphonate, the deprotonated oxygen of the phosphonate acts as the acceptor in a hydrogen bond with the amine of K34, which coordinates to a water molecule enclosed in the receptor’s active site. The phosphoryl oxygen forms one hydrogen bond with the protonated secondary amine of the residue of R120^3.28^ and another intramolecular hydrogen bond with the ligand’s own protonated amine. The C8 chain of fingolimod-*(R)*-phosphonate inserts between residues F265^6.44^ and W269^6.48^, and also appears to align with S304^7.46^ in the transmembrane domain of S1P_1_.

### Binding of FTY720-*(S)*- and *(R)*-Enephosphonates to S1P_1_

During synthesis of the fingolimod phosphonate analogs, vinyl- or enephosphonate intermediates produced noticeable bioactivity (Lu et al., 2009). We also examined the binding behavior of these previously described *(S)*- and *(R)*-enephosphonate analogs of FTY720 to S1P_1_.

**FIGURE 7:**
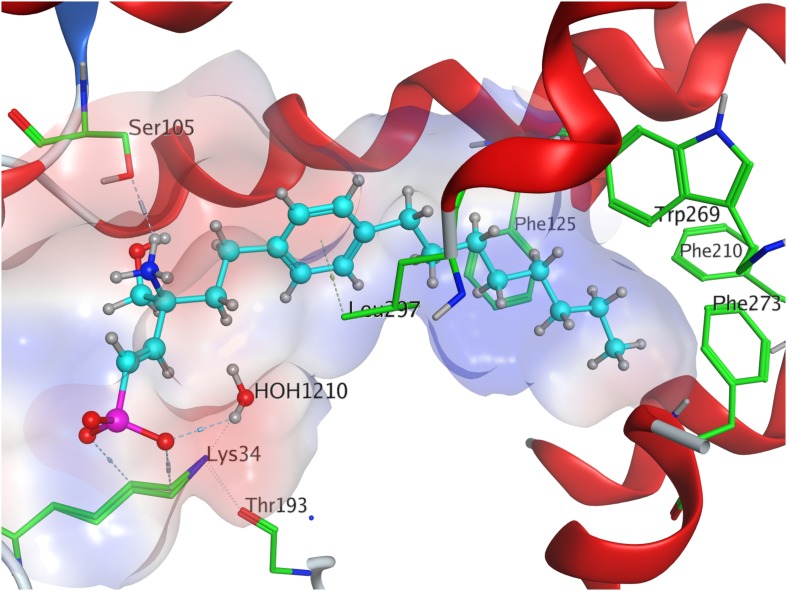
Energetically lowest calculated conformation of FTY720-*(S)*-enephosphonate in S1P_1_.

Docking of the *(S)*-enephosphonate derivative of fingolimod yielded a minimal energy of conformer value of −314.3 kcal/mol (Table 3). Up to the 24th following conformer no greater energy value than −290 kcal/mol is recorded; however, the 25th and final energetically favorable conformer displays a significant increase to −232.5 kcal/mol, possibly indicating conformational limitation through straight due to given unsaturation (see Supplementary Material). In the binding of the *(S)*-enephosphonate, K34 appears to play a significant role, as it forms a coordinating triangle between one deprotonated oxygen atom of the phosphonate and a trapped water molecule inside the active site’s cavity of S1P_1_, which, in turn, also is coordinated to the phosphonate oxygen. Residue K34 is further fixed by the phosphoryl oxygen of the ligand, as well as the carbonyl of T193. The protonated amine of the ligand forms a hydrogen bond with the deprotonated carboxylic acid of residue E294^7.36^ as well as the oxygen of S105^2.64^. With the aromatic core interacting with L297^7.39^, the alkyl tail resides in a pocket made of aromatic transmembrane residues F125^3.33^, F210^5.47^, F273^6.52^, and W269^6.48^ (Figure 7).

FTY720-*(R)*-enephosphonate was docked to S1P_1_ with obtaining a minimal energetic conformer value of −317.1 kcal/mol (Table 3). The increase in energy values for the remaining 24 conformers recorded was comparably small, as the highest registered energy value measured −291.4 kcal/mol. Interestingly, there was no rapid energy increase in any of the final recorded conformations as seen in the *(S)*-enephosphonate (see Supplementary Material). As observed in its enantiomer, the *(R)*-vinylphosphonate exhibits the same hydrogen bond triangle interaction between the deprotonated oxygen atoms of its phosphonate moiety, residue K34, and a trapped water molecule within the active site of the receptor. What was not observed in the *(S)* enantiomer, however, was the interaction of the C2 vinyl proton of the substrate with the deprotonated carboxylic acid of E294^7.36^ which points directly at it. Furthermore, the enephosphonate’s hydroxyl group donates to a hydrogen bond to S105^2.64^. The hydrophobic tail of the ligand inserts in the aromatic pocket formed by F125^3.33^, F210^5.47^, F273^6.52^, and W269^6.48^.

## Discussion

**FIGURE 8:**
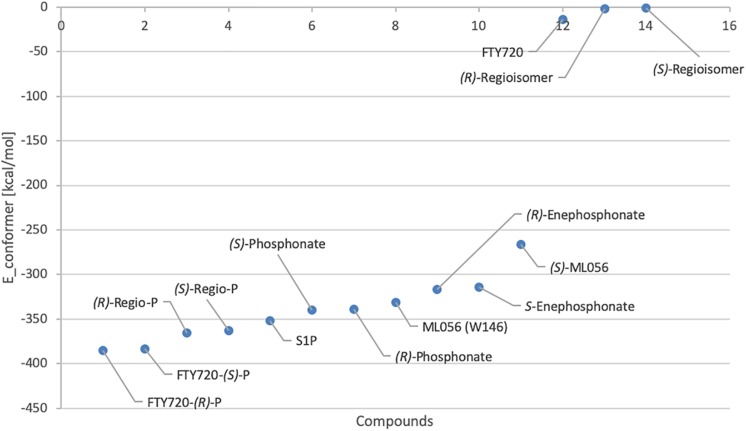
Plot of relative energy of conformer values in (kcal/mol) of docked substrates.

Effective binding of a substrate to S1P_1_ requires polar interactions between a ligand’s hydrophilic head group and the residues located in the receptor’s active site, while occupation of the inter-helical transmembrane domain of the receptor dictates agonism or antagonism (Davis et al., 2007; Troupiotis-Tsaïlaki et al., 2017). Determining trends and similarities between similarly acting substrates helps to understand what structural features synthetic sphingoid analogs need to possess to specifically and agonistically – or antagonistically – target S1P_1_ (Figure 8). Table 4 summarizes all observed positive interactions between all substrates and the specific residues of S1P_1_.

**TABLE 4:** Interaction summary between docked substrates and specific residues of S1P_1_. Each × marks an attracting interaction between respective residue and substrate.

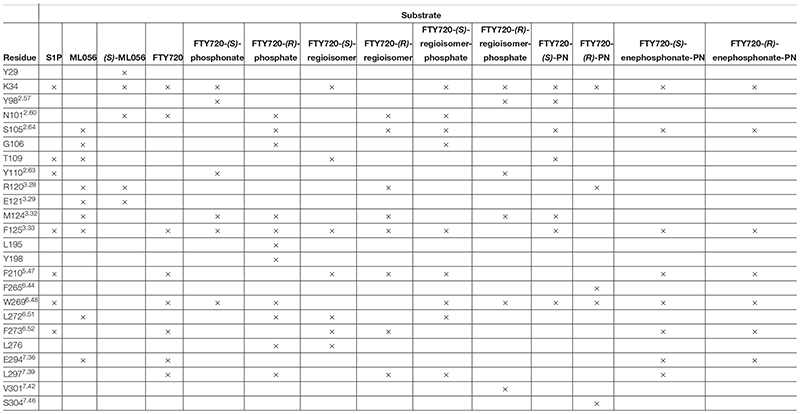

### Polar Head Group Interactions

The comparison between phosphorylated and unphosphorylated substrates illustrates the relevance of phosphorylation of the ligand for high–affinity interaction with S1P_1_; the considerable differences between calculated energy of conformer values of unphosphorylated substrates and their respective phosphates and phosphonates result from a multitude of additional polar interactions observed only upon presence of a phosphate or phosphonate head group and a zwitterionic character of the overall ligand (Hanson et al., 2012). Residues K34, S105^2.64^, Y110^2.63^, and T109 play a substantial role in fixation of a phosphate within the polar active cavity facing the extracellular compartment of S1P_1_. Analyses of conformations of S1P, the phosphate enantiomers of fingolimod, as well as the respective regioisomer phosphates within the active site of the receptor strongly suggest that the hydroxyl groups of T109 and Y110^2.63^ effectively engage in hydrogen bonding with the deprotonated oxygen atoms and the phosphoryl oxygen of the phosphoester, locking the head group in place and inducing the binding event. The protonated amino group of K34 appears to be of essential importance, as nearly all docking models showed strong and often multiple intramolecular interactions with the side chain and terminal amine of this lysine residue; the phosphoryl oxygen of the ligands’ phosphate or phosphonate moieties showed particular affinity to K34, often forming a bridged triangle with one water molecule trapped inside the active cavity of S1P_1_. This presence of water is assumed to be crucial for proper binding functionality of substrates, as previous library screenings of class II agonists indicated that it is mainly K34 that continuously binds to the substrate’s phosphate, whereas polar residues N101^2.60^, R120^3.28^, and E121^3.29^ thought to be essential for binding actually do not engage the phosphate itself (Gonzalez-Cabrera et al., 2008; Schürer et al., 2008; Yuan et al., 2013). S105^2.64^ was also shown to interact with the protonated amino groups of fingolimod phosphates and phosphonates, but also acted as a donor for hydrogen bonding with the hydroxyl group of phosphonate substrates, as previously calculated in distinct MD simulations by Yuan et al. (2013).

In contrast to the high-affinity binding of phosphorylated ligands, unphosphorylated substrates showed relatively high conformational energy values when docked to S1P_1_, indicating high *K*_*d*_ values of these complexes (Figure 8 and Table 3). K34 and E294^7.36^ (in the case of unphosphorylated fingolimod), S105^2.64^ and R120^3.28^ (unphosphorylated *(R)*-regioisomer), and T109 (for the unphosphorylated *(S)*-regioisomer) were the only residues that displayed attractive interactions with the hydroxyl groups and the protonated amine, but lacked consistency in the optimized conformers recorded. As extracellular loops 1 (ECL1) and 2 (ECL2) of S1P_1_ are known to pack over the *N*-terminus of the receptor, folding over the amphipathic groove on the external ligand binding site, substrates that lack high affinity to respective active cavity do not enter and stay within the receptor, failing to bind and activating it (Rosen et al., 2009).

### Lipophilic Tail Interactions

While the polar active site serves as the inducing binding event of a substrate to S1P1, the interaction of the lipid tail of the ligand with the hydrophobic transmembrane domain of the receptor determines agonism versus antagonism (Truc-Chi et al., 2008; Yuan et al., 2013; Troupiotis-Tsaïlaki et al., 2017). From the crystal structure of the S1P_1_ in conformation with antagonist ML056 (W146) and from previous MD experiments with S1P, it is concluded that the hydrophobic tail of potential S1PR ligands inserts into a mostly aromatic binding pocket formed by residues from the transmembrane helices TM3, TM5, TM6, and TM7; in particular, residues F125^3.33^, F210^5.47^, and F273^6.52^ form a cluster centered around TM5 that is highly responsive to the shape and length of a substrate’s lipid tail (Hanson et al., 2012; Yuan et al., 2013; Troupiotis-Tsaïlaki et al., 2017). This observation was confirmed in our docking experiments; the C8 alkyl chains of all docked substrates, including unphosphorylated compounds, were consistently found embedded between phenylalanine residues F125^3.33^, F210^5.47^, and F273^6.52^. Interestingly, residues N101^2.60^ and L297^7.39^ often interacted with the aromatic core of the substrates rather than with the polar head groups. Residue M124^3.32^ was also found to align regularly with the often-bent lipid tail of the fingolimod phosphates and derivatives, contributing to a lipophilic binding pocket. Yuan et al. (2013) postulated the occupation and movement of the sphingoid acyl tail results in flipping of reside W269^6.48^, which, in turn, induces water influx into the receptor by rearrangement of helix TM6, activating a conformational switch for binding of a G protein from the cytoplasmic site. Without exception, all substrates subjected to S1P_1_ docking displayed alignment and rearrangement of W269^6.48^ in proximity to the aromatic pocket, strengthening the assumption that insertion into the phenylalanine pocket between TM3, TM5, TM6, and TM7 does induce a rearrangement of the tryptophan residue. However, for further analyses of the exact mechanism of activation of S1P_1_ through agonism a different simulation might be required, as the occupation of S1P_1_ by a sole agonist in a microenvironment without a coupled G protein cannot induce and stabilize the fully active state of the receptor (Nygaard et al., 2013; Troupiotis-Tsaïlaki et al., 2017). With the hydrophobic pocket formed by F125^3.33^, F210^5.47^, and F273^6.52^ and a fixed aromatic center by interacting residues N101^2.60^ and L297^7.39^, the substitution pattern around the benzene ring is expected to define the bioactivity of the substrate; as fingolimod and its *para* analogs are considered agonists, *para* substitution is associated with agonism, while *meta* substitution (as seen in ML056/W146) indicates antagonism due to distortion of the transmembrane helices through the phenylalanine binding pocket associating around the lipid tail (Hanson et al., 2012; Troupiotis-Tsaïlaki et al., 2017).

### Stereochemical and Intramolecular Effects

Both enantiomers of all chiral substrates were subjected to the identical docking simulation to elucidate possible preferences regarding the stereochemistry of the compounds. Interestingly, with only one exception, we found that in all cases the *(R)* enantiomer showed slightly more favorable binding in the *in silico* docking experiments (Figure 8 and Table 3). The smallest difference in energy values could be recorded during docking of the FTY720-*(S)*- and *(R)*-phosphonates; the optimized energy of the most stable conformer of the *(R)* enantiomer in S1P1 was found to be 1.319 kcal/mol lower than the one of its *(S)* counterpart. The *(R)*-phosphate of FTY720 achieved a conformer with a potential energy lower by 1.567 kcal/mol in comparison with its *(S)* enantiomer, the phosphates of the fingolimod regioisomer differ by 2.642 kcal/mol in favor for the *(R)* enantiomer, and the E-vinylphosphonate enantiomers show a energetical difference of 2.785 kcal/mol with the *(R)* enantiomer being the energetically more favorable conformer.

The docked unphosphorylated regioisomers of fingolimod displayed an energetical difference of 1.103 kcal/mol, being the lowest discrepancy of all recorded chiral substrates in this experiment, indicating the lack of relevance of chirality upon low binding affinity due to the absence of a phosphate moiety. This insinuates it is predominantly the phosphorous–carrying group of the substrate whose symmetry and three-dimensional orientation does make a difference in preferential binding, even if that difference appears to be subtle in *in silico* experiments. Since the amino group and the hydroxyl moiety of the ligands are bound to the same chiral carbon, hence, inevitably point in opposite directions, it is the presence, orientation, and intra- as well as intermolecular interaction of the third moiety (the phosphate or phosphonate) that locks the substrate in a specific conformation. The phosphate (phosphonate) effectively inhibits the molecule’s free rotation along its own axis so it could always meet an optimal pose for its amino and hydroxyl groups to freely match with residues on opposite sides of the cavity. This is achieved by both intermolecular as well as intramolecular interactions of the phosphate (phosphonate); while anchoring the substrate predominantly to K34, S105^2.64^, and Y110^2.63^ (intermolecularly), the phosphates and phosphonates develop in all cases intramolecular hydrogen bonds with majorly the ligand’s own protonated amino group. This rigidity locks a sterically fitting substrate along with the intermolecular interactions of its functional groups oriented toward matching residues in a position where the hydrophobic alkyl tail can insert between the transmembrane helices, being embedded in the aromatic binding pocket, and induce a rearrangement of the transmembrane helices, eventually leading to the activation of the receptor and its interaction with a coupled G protein.

## Conclusion

Examining the binding behavior of S1P and the sphingoid derivative, fingolimod, its phosphate enantiomers, phosphonate derivatives, and regioisomers to S1P_1_ via *in silico* docking simulations using MOE^®^ visualized the relevance of phosphorylation for sphingosine-derived S1PR modulators, showed which residues within the active site of the receptor majorly contribute to the fixation of the substrate, and confirmed the formation of a hydrophobic binding pocket consisting of mostly phenylalanine residues between transmembrane helices TM3, TM5, TM6, and TM7. K34 has proven to be of major impact of holding a substrate by its phosphate or phosphonate moiety in place, supported by an enclosed water molecule that often acts as a bridge between the amine of the ligand or other residues within the active site. Depending on the substrate, S105^2.64^, G106, T109, and Y110^2.63^ also hydrogen-bind to the oxygen of the phosphorous–carrying moiety. The aromatic center of the molecule tends to be locked between N101^2.60^ and L297^7.39^, though this was not observed continuously in every ligand docked to the receptor. The insertion of the substrate’s alkyl tail into a cluster comprised of F125^3.33^, F210^5.47^, and F273^6.52^, often causing a rearrangement of W269^6.48^, was confirmed in all phosphorylated ligands, highlighting the functionality of these hydrophobic residues for receptor activation. This confirms that the actual activation of the receptor is distinct from the initial binding event of the substrate to the active site.

With the phosphate/phosphonate moieties strongly interacting intramolecularly with the amino group and occasionally with the hydroxyl moiety, the ligand is rigidified within the microenvironment of the active cavity, which facilitates its locking into position if sterically favorable. This is being influenced by the ligand’s stereochemistry which reflects in the slight energetical preference of *(R)* enantiomer conformers binding to S1P_1_ over the respective *(S)* isomers (Figure 8).

While enhanced binding affinity determination *in silico* to a certain receptor, as S1P_1_ in this case, indicate greater specificity for respective receptor, the eventual bioactivity of synthetic drug candidates continues to require *in vitro* and *in vivo* experiments to observe the effects of the compounds of interest not only in a microcosmos of a simulation, but in the complex macroscopic environment of a living organism.

## Data Availability Statement

All datasets generated for this study are included in the article/Supplementary Material.

## Author Contributions

All authors wrote the manuscript. AM performed the calculations.

## Conflict of Interest

The authors declare that the research was conducted in the absence of any commercial or financial relationships that could be construed as a potential conflict of interest.

## Footnotes

1 Chemical Computing Group official website, URL: https://www.chemcomp.com/Products.htm (accessed: 07/16/2019).

2 ERRAT validation UCLA website, URL: https://servicesn.mbi.ucla.edu/ERRAT/ (accessed: 02/01/2020).
