# Supplementary material for: In silico Docking Studies of Fingolimod and S1P1 Agonists
Source: Front Pharmacol. 2020 Mar 10;11:247. doi: 10.3389/fphar.2020.00247 (PMC7076195; doi:10.3389/fphar.2020.00247)
Supplement: Supplementary file 1 [file Data_Sheet_1.docx]

*In Silico* Docking Studies of Fingolimod and S1P_1_ Agonists

Alexander Marciniak^1^, Sara M. Camp^2^, Joe G. N. Garcia^2^, Robin Polt^1*^

^1^*Department of Chemistry & Biochemistry, The University of Arizona, Tucson, AZ 85721, USA*

^2^*Department of Medicine, The University of Arizona, Tucson, AZ 85724, USA*

*** Correspondence:**Robin Polt
polt@u.arizona.edu

# Supplemental Material Figures

**
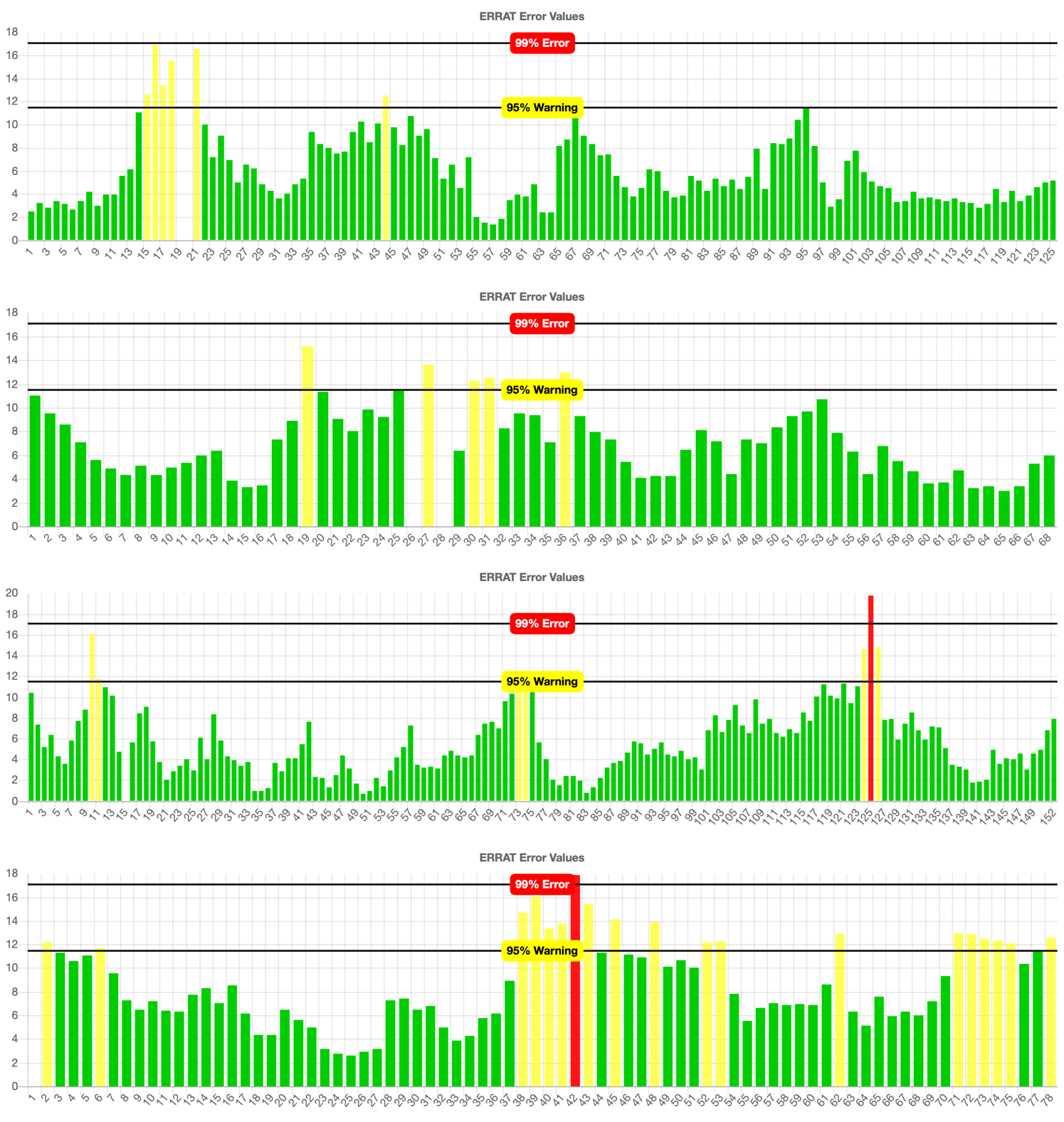
**

**Figure S1.** All four ERRAT plots (from top to bottom: Quality Factor plots A, B, C, D) as obtained from crystal structure data validation of S1P_1_ extracted from PDB file 3V2Y (Hanson *et al.,* 2012)^[[1]](#footnote-1)^. Quality Factor (QF) A: 95.1%; QF B: 92.4%; QF C: 95.4%; QF D: 75.3%.


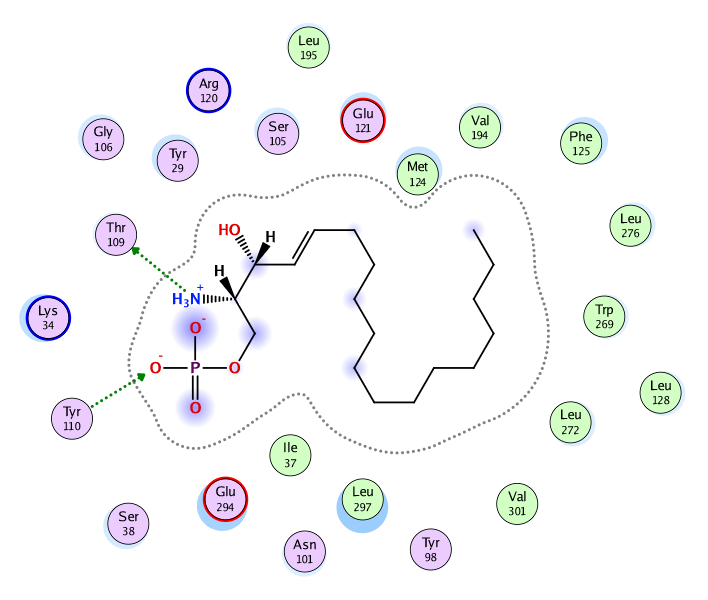


**Figure S2.** 2D depiction of binding interactions of S1P in S1P_1_. Polar residues of the polar active site of S1P_1_ are shown purple, nonpolar residues appear as green. Dotted green arrows mark the donation to hydrogen bonding.


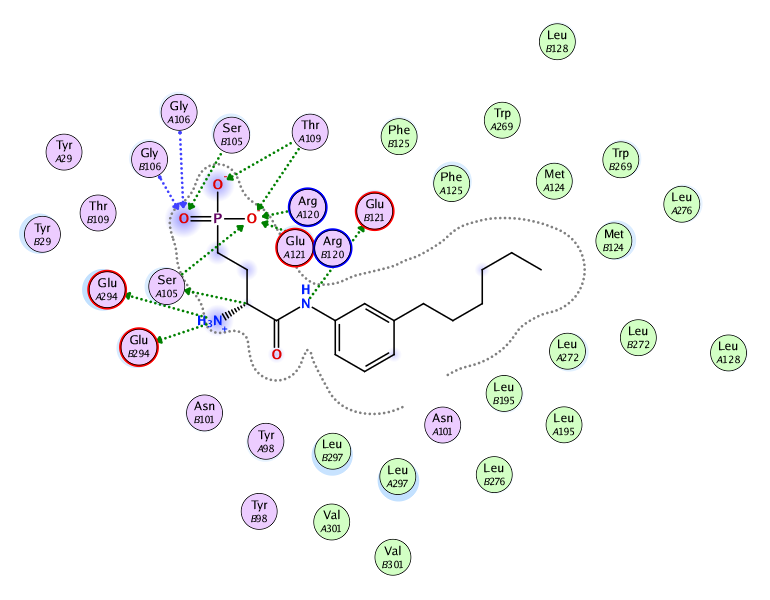


**Figure S3.** 2D depiction of binding interactions of ML056 (W146) in S1P_1_. Polar residues of the polar active site of S1P_1_ are shown purple, nonpolar residues appear as green. Dotted green arrows mark the donation to hydrogen bonding.

**
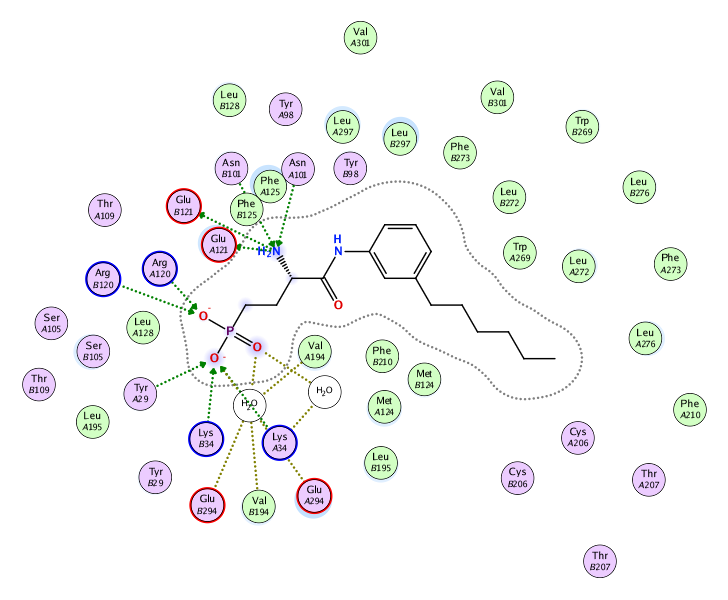
**

**Figure S4.** 2D depiction of binding interactions of *(S)*-ML056 in S1P_1_. Polar residues of the polar active site of S1P_1_ are shown purple, nonpolar residues appear as green. Dotted green arrows mark the donation to hydrogen bonding. Note obstruction of polar interactions between the protonated amino group of the substrate and N101^2,60^ and E121^3,29^ by F125^3,33^.

**
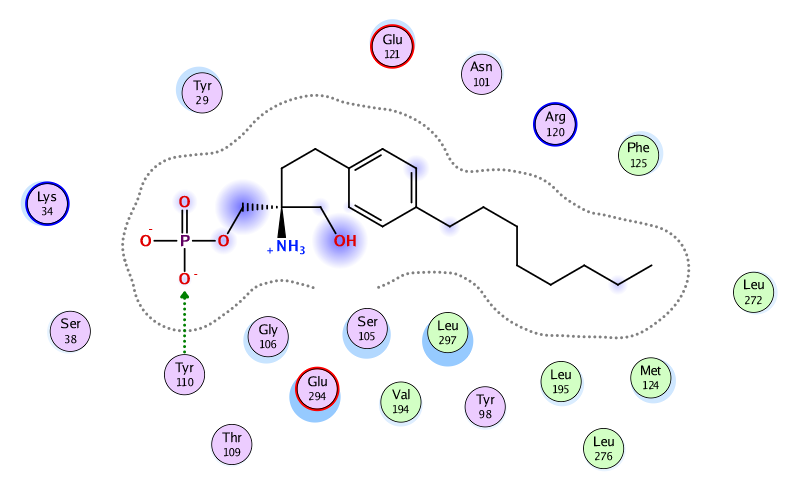
**

**Figure S5.** 2D depiction of binding interactions of FTY720-*(S)*-phosphate in S1P_1_. Polar residues of the polar active site of S1P_1_ are shown purple, nonpolar residues appear as green. Dotted green arrows mark the donation to hydrogen bonding.

**
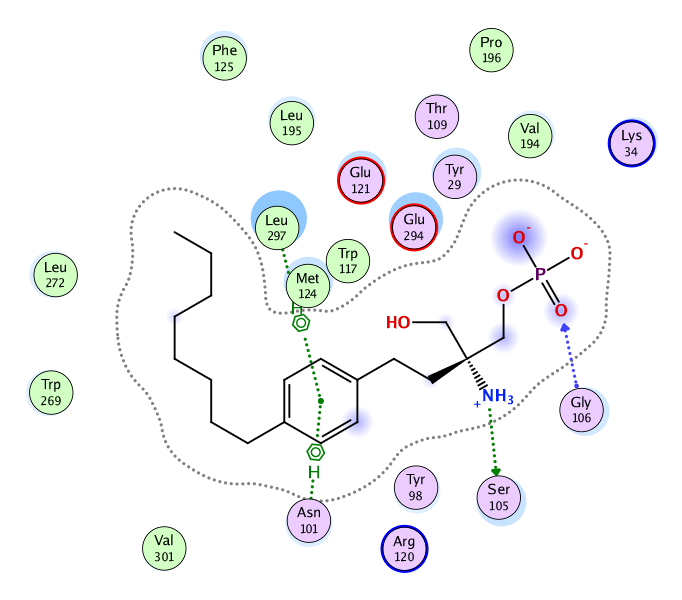
**

**Figure S6.** 2D depiction of binding interactions of FTY720-*(R)*-phosphate in S1P_1_. Polar residues of the polar active site of S1P_1_ are shown purple, nonpolar residues appear as green. Dotted green arrows mark the donation to hydrogen bonding. Aromatic interactions are marked with an aryl label.

1. ERRAT Validation UCLA Website, URL: *https://servicesn.mbi.ucla.edu/ERRAT/* (accessed: 02/01/2020). [↑](#footnote-ref-1)
